# Efficacy and Safety of Chimeric Antigen Receptor T Cells in Acute Lymphoblastic Leukemia With Post-Transplant Relapse

**DOI:** 10.3389/fonc.2021.750218

**Published:** 2021-11-01

**Authors:** Lijuan Ding, Yiyun Wang, Ruimin Hong, Houli Zhao, Linghui Zhou, Guoqing Wei, Wenjun Wu, Huijun Xu, Yanlei Zhang, Yi Luo, Jimin Shi, Alex H. Chang, Yongxian Hu, He Huang

**Affiliations:** ^1^ Bone Marrow Transplantation Center, First Affiliated Hospital, College of Medicine, Zhejiang University, Hangzhou, China; ^2^ Liangzhu Laboratory, Zhejiang University Medical Center, Hangzhou, China; ^3^ Institute of Hematology, Zhejiang University, Hangzhou, China; ^4^ Zhejiang Province Engineering Laboratory for Stem Cell and Immunity Therapy, Hangzhou, China; ^5^ Shanghai YaKe Biotechnology Ltd, Shanghai, China

**Keywords:** chimeric antigen receptor T cells, post-transplant relapse, acute graft versus host disease, cytokine release syndrome, acute lymphoblastic leukemia

## Abstract

Twenty patients with relapsed B-ALL after HSCT were treated with CAR T cell therapy and were evaluated for efficacy and safety. Twelve patients previously received haploidentical transplantation, while 8 patients received HLA-matched transplantation. The median relapse time was 12 months (range, 4 to 72). Thirteen patients received autologous CAR T cells, and 7 patients received allogeneic CAR T cells, which were derived from transplant donors. The median infusion dose was 2.9×106/kg (range, 0.33 to 12×106/kg). Nineteen patients were evaluated for efficacy, among which 17 patients (89.5%) achieved MRD negative. The CR rates in the HLA-matched transplantation group and haploidentical transplantation group were 100% (7/7) and 83.3% (10/12), respectively. The median follow-up time was 9.80 months (range, 2.40 to 64.97). Ten patients (50%) died of relapse, 3 patients (15%) died of infection, and 1 patient (5%) died of aGVHD. Fifteen patients (75%) developed CRS, including 3 (20%) grade 1 CRS, 6 (40%) grade 2 CRS, and 6 (40%) grade 3 CRS. Ten patients (50%) developed aGVHD, including 1 (10%) grade I aGVHD, 6 (60%) grade II aGVHD, and 3 (30%) grade III aGVHD. The log rank test showed that CAR T cell origin was correlated with aGVHD occurrence in the haploidentical transplantation group (P = 0.005). The authors’ study indicated that the initial efficacy and safety of CAR T cell therapy for patients with post-transplant relapse were satisfactory. However, aGVHD was a concern in patients with a history of haploidentical transplantation occupied with allogeneic CAR T cells, which warrants clinical attention.

## Introduction

The prognosis of relapsed acute lymphoblastic leukemia (ALL) after hematopoietic stem cell transplantation (HSCT) is dismal. A retrospective study of 465 cases of relapsed ALL after 1^st^ HSCT showed that the median survival time was only 5.5 months. The 1-year, 2-year, and 5-year survival rates were 30 ± 2%, 16 ± 2% and 8 ± 1%, respectively (1). A study of 232 pediatric patients showed that the 3-year overall survival (OS) after relapse was 13% (2). For post-transplant relapses, donor lymphocyte infusion (DLI), second transplantation, intense chemotherapy and cytokine treatment have been tried as salvage regimens without significant clinical benefits. The success of DLI varies from 0 to 57.1% with mere increase in median survival by 6 months (3, 4). Noteworthy, treatment-related mortality after DLI is 5-20% and more than one-third of patients will develop acute and/or chronic GVHD after DLI (5). Second transplantation resulted in an OS of only 14% at 2 years among 245 patients (6). The poor performance and high expenditure of second transplantation have made it an unwise choice for post-transplant relapses. Failure of the above regimens highlights the need for novel alternative and more effective therapies.

In recent years, chimeric antigen receptor (CAR) T cell therapy has yielded pivotal success in hematological disease, especially in refractory/relapses (R/R) ALL. Autologous CD19 CAR T cells have shown significant antileukemia effects, with complete remission (CR) rates of 70~90% (7–10). The cumulative incidence of relapse (CIR) and disease-free survival (DFS) brought by CAR T cell therapy itself are relatively short. Therefore, successful CAR T cell therapy are often followed by allo-HSCT. However, limited studies have focused on CAR T cells in the treatment of post-transplant relapses. For patients with a history of allo-HSCT, the source of CAR T cells can be either the patient himself or the transplant donor, which is called allogeneic CAR T cells in this manuscript. Preclinical studies demonstrated that donor-derived CAR T cells were active in allogeneic recipients but had the capacity to induce lethal GVHD (11). Hu Y et al. previously reported a retrospective comparison of allogenic and autologous CD19 CAR T cell therapy in 31 ALL patients, including 14 patients with post-transplant relapses (12). Univariate subgroup analysis of allogenic CAR T group showed the presence of cGVHD at the time of T cell collection was significantly associated with less than 6-month relapses (*P* = 0.022). aGVHD occurred in 3 patients with post-transplant relapses.

However, the long-term efficacy and safety of CAR T cells in the treatment of post-transplant relapses have not yet been systematically clarified. Here we report the retrospective clinical results of CAR T cell therapy in 20 patients with relapsed ALL after HLA-matched or haploidentical transplantation from our hospital.

## Methods

### Patients and Study Design

**Figure 1:**
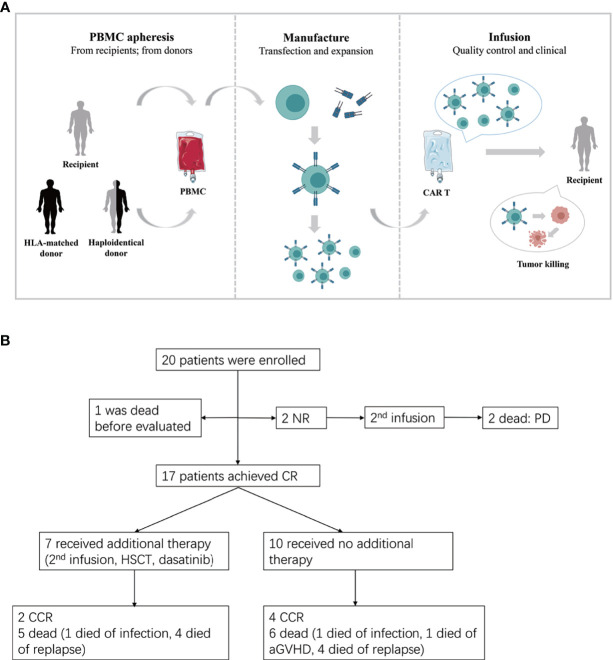
**(A)** The procedure of autologous or allogeneic CAR T cells in patients with post-transplant relapse. Peripheral blood mononuclear cells (PBMC) are apheresised from recipients or transplantation donors (including HLA-matched donor and haploidentical donor). CD3 positive T cells are selected and stimulated by CD3/CD28 dynabeads. Then T cells were transfected by lentivirus to generate CAR T cells. Lastly, the qualified CAR T cells are infused to recipients after expansion. **(B)** Study flow, enrollment, treatment and follow-up of patients. CR, complete remission; CCR, continued CR; NR, non-remission; PD, progressive disease.

Twenty patients with relapsed ALL after HLA-matched or haploidentical transplantation were enrolled in this retrospective study (**Figure 1B**) and were administered CAR-T cell therapy at the First Affiliated Hospital of Zhejiang University from August 2015 to September 2020. All these patients had received at least one transplantation and remained CR for a period of time afterwards. However, there were no effective regimens when they relapsed after transplantation. None of the patients had been treated with cell-immunotherapy, except one patient who received the first CAR T cell therapy before allo-HSCT. The clinical data were extracted from the electronic medical record system of patients enrolled in the trial ChiCTR-ORN-16008948 and ChiCTR1900023957. The study was approved by the Ethics Committee of the First Affiliated Hospital of Zhejiang University School of Medicine and was conducted in accordance with internationally recognized good clinical practice guidelines.

### CAR T Cell Generation

The protocol of CAR-T cell generation in our center was described previously (12). Briefly, CD3^+^ T cells were separated from peripheral blood mononuclear cells and then transduced with a lentiviral vector containing a CAR with a CD3-zeta domain and 4-1BB domain. CAR T cells were expanded for another 5 days until the numbers were sufficient for infusion (**Figure 1A**).

### Treatment and Detection

All patients received conditioning chemotherapy with concurrent cyclophosphamide (500-750 mg/m^2^) and fludarabine (30-50 mg/m^2^) administration for 3 days followed by CAR T cell infusion. CRS and aGVHD prevention were not performed. The classification of CRS and aGVHD refer to the standard (13, 14). Bone marrow was evaluated in 1 month after the initial infusion by standard pathological testing. Then patients were followed-up in the clinic every three months.

### Statistical Analysis

Measurement data were described using means with standard deviations or medians with ranges. Enumeration data are presented as frequencies (%). Kaplan-Meier (KM) curves for PFS were generated, and the log-rank test was used to compare differences between subgroups. The median follow-up time was estimated using reverse Kaplan-Meier curves. All *P* values represented were two-sided, and results were considered statistically significant when *P* < 0.05 (*). Data were analyzed using IBM SPSS Statistics 24. Prism (version 7.0) was used to generate the graphs.

## Results

### Patient Characteristics

The 20 patients were evaluated for efficacy and safety. The characteristics of the patients are shown in **Table 1**. Ten patients were male. Median age was 27 years old (range, 15 to 66). The proportion of Ph+ ALL was 15% (3/20). Twelve patients previously received haploidentical transplantation, and 8 patients received HLA-matched transplantation. The median relapse time after transplantation was 12 months (range, 4 to 72). Further, 13 patients received autologous CAR T cells, and 7 patients received allogeneic CAR T cells. Allogeneic CAR-T cells were manufactured from transplant donors, which were adopted for cases where there were insufficient or deficient autologous T lymphocytes. The median infusion dose was 2.89×10^6^/kg (range, 0.33 to 12×10^6^/kg). All the patients were enrolled in the CAR-T treatment trial only after they had stopped chemotherapy for more than 1 month. There were no statistically significant differences in sex, age, disease karyotype, or leukemia burden.

**Table 1:** Patients baseline and therapy-related characteristics.

**Gender, no. (%)**	
Female	10 (50)
Male	10 (50)
**Age, range (year)**	27 (15-66)
**Hyperploidy, n (%)**	1 (5)
**Hypoplodiy, n (%)**	0 (0)
**Complex karyotype, n (%)**	2 (10)
**BCR-ABL1, n (%)**	5 (25)
**ETV6-RUNX1, n (%)**	0 (0)
**E2A-PBX1, n (%)**	0 (0)
**KMT2A rearranged, n (%)**	1 (5)
**IKZF1 mutation, n (%)**	0 (0)
**Poor-risk cytogenetics, n (%)**	4 (20)
**Good-risk cytogenetics, n (%)**	1 (5)
**Leukemia burden, range (%)**	11.96 (0-83)
**Prior numbers of therapy, range(no.)**	4 (1-9)
**Numbers of relapses, range (no.)**	1 (1-3)
**Transplantation type, no. (%)**	
Matched transplantation	8 (40)
Haploidentical transplantation	12 (60)
**Relapse time after transplantation, range (month)**	12 (4-72)
**CAR T-cell dose, range (×10^6^/kg)**	2.89 (0.33-12)
**CAR T origin, no. (%)**	
Autologous	13 (65)
Donor	7 (35)

### Efficacy

Nineteen of the 20 patients survived for more than one month were evaluated for efficacy, except one patient who died of severe lung infection within one week. Among the19 patients, 17 patients achieved MRD negative in one month confirmed by bone marrow examination. The complete remission (CR) rate was 89.5%. The CR rates in the HLA-matched transplantation group and haploidentical transplantation group were 100% (7/7) and 83.3% (10/12), respectively. Considering CAR T cell origin, The CR rates of allogeneic CAR T cells and autologous CAR T cells were 85.7% (6/7) and 91.7% (11/12), respectively. More specifically, the CR rates of allogeneic CAR T cells and autologous CAR T cells in the HLA-matched transplantation group and haploidentical transplantation group were 100% (3/3), 100% (4/4), 87.5% (7/8) and 75% (3/4). Due to the lack of close monitoring in the preliminary clinical trials, the CAR-T cell expansion *in vivo* were not detected continuously, which made it impossible to learn the CAR T cell dynamics in different groups.

**Figure 2:**
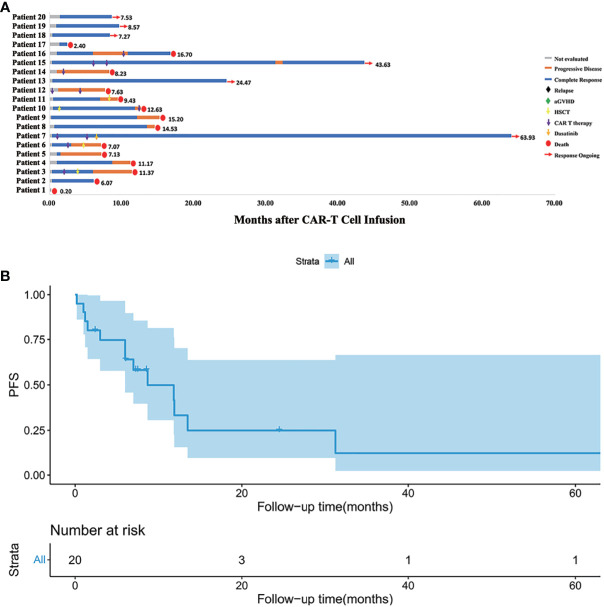
**(A)** Follow-up results of each patient. Patient 1 died of severe lung infection within one week after CAR T cell infusion. Patients 12 and 14 progressed even after second CAR T cell infusion. Patient 7 with extramedullary relapse maintained CR with dasatinib therapy. Patients 13, 15, 18, 19, 20 maintained CR for now by CAR T cell therapy and chemotherapy. Other patients eventually died of relapse or severe complication. **(B)** Kaplan-Meier (KM) curves for PFS. The median PFS time was 8.7 months, and the 1-year PFS was 33.2%.

Treatment and the response duration of each patient was shown in **Figure 2**. The median follow-up time was 9.80 months (range, 2.40 to 64.97). The median follow-up time in the HLA-matched transplantation group and haploidentical transplantation group were 8.57 months (range, 6.07 to 37.67) and 11.90 months (range, 2.40 to 64.97). There were 10 patients died of relapse or progressive disease, 3 patients died of infection, and 1 patient died of aGVHD. Six patients survived before this manuscript. Patient 7 with extramedullary relapse received two more CAR T cell therapies and used dasatinib as a maintenance therapy (15). Patients 13, 15, 18, 19 and 20 maintained CR by CAR T cell therapy and chemotherapy.

### Toxicitiy

#### CRS

**Figure 3:**
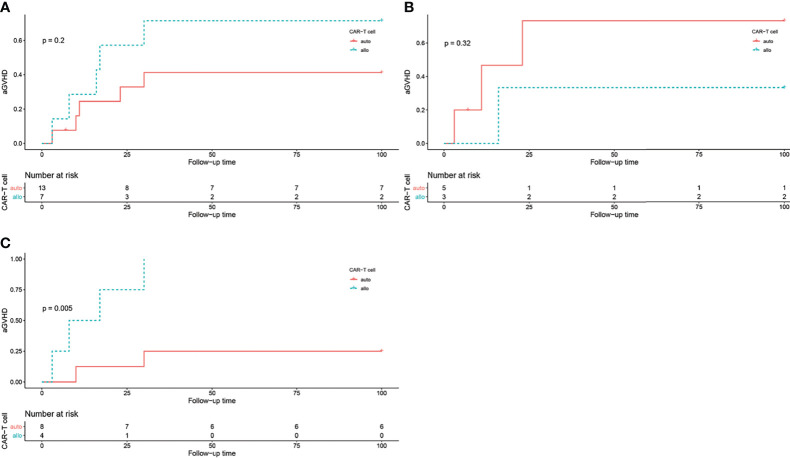
The log rank test of aGVHD incidence in different groups, grouped by CAR T cell origin. **(A)** aGVHD incidence in all patients, P = 0.2. **(B)** aGVHD incidence in all patients in HLA-matched transplantation group, P = 0.32. **(C)** aGVHD incidence in all patients in haploidentical transplantation group, P = 0.005.

Fifteen patients (75%) developed different degrees of CRS, including 3 cases (20%) of grade 1 CRS, 6 cases (40%) of grade 2 CRS, and 6 cases (40%) of grade 3 CRS. No grade 4 and grade 5 CRS occurred. The incidence rates of different grades of CRS were shown in **Figure 3**. One patient had neurological symptoms of epilepsy, elevated cerebrospinal fluid cytokines, and severe CRS. CRS and neurological symptoms in 15 patients lasted for a short time and were quickly controlled after hormone therapy. No patients died directly of CRS or neurotoxicity.

#### aGVHD

Allogeneic CAR T cells brings the concern of aGVHD. In our study, 10 (50%) patients developed the symptoms of aGVHD, including 1 (10%) grade I aGVHD, 6 (60%) grade II aGVHD, and 3 (30%) grade III aGVHD. Among them, the aGVHD incidence rates in the HLA-matched transplantation group and the haploidentical transplantation group were 50% (4/8) and 50% (6/12), respectively (*P* = 0.725, **Table 2**). Benefiting from our clinical experience in the treatment of aGVHD after allo-HSCT, the symptoms were effectively controlled by immunosuppressants, which include etanercept, cyclosporine, tacrolimus and methylprednisolone.

**Table 2:** Univariate analysis of possible factors related to aGVHD.

Factors	No. of patients (%)	No. of aGVHD (%)	*P* value
**Overall**	20 (100)	10 (50)	
**Gender**			0.464
Male	10 (50)	6 (60)	
Female	10 (50)	4 (40)	
**CAR T cell source**			0.22
Autologous	13 (65)	5 (38.5)	
Allogeneic	7 (35)	5 (71.4)	
**History of DLI**			0.25
Yes	6 (30)	5 (83.3)	
No	14 (70)	5 (35.7)	
**Transplantation type**			0.725
Matched transplantation	8 (40)	4 (50)	
Haploidentical transplantation	12 (60)	6 (50)	
**aGVHD post-HSCT**			0.534
Yes	8 (40)	3 (37.5)	
No	12 (60)	7 (58.3)	
**Duration of CR post-HSCT (months)**			0.198
0-6	6 (30)	5 (83.3)	
7-12	5 (25)	1 (20)	
>12	9 (45)	4 (44.4)	
**CRS grade**			0.487
0-2	14 (70)	7 (50)	
3	6 (30)	3 (50)	
**Agranulocytosis time (days)**			0.373
0-14	15 (75)	8 (53.3)	
15-28	4 (20)	2 (50)	
>28	1 (5)	0 (0)	
**Chromosome**			0.659
Ph+	3 (15)	1 (33.3)	
Ph-	17 (85)	9 (62.9)	

In the HLA-matched transplantation group, 3 out of 5 patients (60%) received autologous CAR T cells and 1 out of 3 patients (33.3%) received allogeneic CAR T cells developed aGVHD. While in the haploidentical transplantation group, 2 out of 8 patients received autologous CAR T cells and all 4 patients received allogeneic CAR T cells developed aGVHD. The log-rank test showed that CAR T cell origin was correlated with aGVHD occurrence in the haploidentical transplantation group (*P* = 0.005, **Figure 3**).

## Discussion

Relapse after allo-HSCT has always been a considerable obstacle in the treatment of R/R ALL. The remission rate of second transplantation and DLI treatment are relatively unsatisfactory (3–6). Therefore, new treatment methods are urgently needed in patients with post-transplant relapse. CAR T cell therapy, which has emerged as a promising treatment for hematological malignancies in recent years, has achieved high initial response rates and long-term remission in R/R ALL. However, research of CAR T cells in the treatment of post-transplant relapse is still in its infancy. Brudno et al. used donor-derived CD19 CAR T cells in patients with persistent B-cell malignancies after allo-HSCT. Eight out of 20 patients showed tumor regression without aGVHD flare, including six achieving complete remission (CR) and two achieving partial remission (PR) (16). Maude et al. performed CD19 CAR T cell therapy on 18 patients who relapsed after allo-HSCT. There was no significant difference in terms of event-free survival (EFS) or overall survival (OS) between patients who had received allo-HSCT and those who had not (P = 0.21 for EFS, P = 0.24 for OS) (17). The authors’ study showed a CR rate of 89.5% (17/19). The CR rates of the HLA-matched transplantation group and haploidentical transplantation group were 100% (7/7) and 83.3% (10/12), respectively.

Relapse after CAR T cell therapy remains a big obstacle that influence patients’ efficacy and long-term survival. Most patients relapse within 1 year after CART treatment (18). In the authors’ study, the median relapse time was 6.07 months (range, 1.50 to 31.27). At present, the mechanism of relapse after CAR-T cell therapy is still unclear. According to current studies, CD19-negative relapse is related to the loss of CD19 gene (19) or myeloid transformation (20). The possible mechanism of CD19-positive relapse includes CAR-T cell exhaustion and abnormal CAR-T cell function caused by immunosuppressive cells and factors in the bone marrow microenvironment (such as mesenchymal stem cells (MSCs), regulatory T cells (Treg), bone marrow-derived suppressive cells (MDSC), TGF-β, etc.) (21).

Toxicities after CAR T cell infusion include CRS, tumor lysis syndrome (TLS), immune effector cell-associated neurotoxicity syndrome (ICANS) and B-cell aplasia. aGVHD was brought to concern when allogeneic CAR T cells were applied. aGVHD occurs when the T lymphocytes in allogeneic donor transplants attack the recipient’s target cells, which is stimulated by a series of “cytokine storms” initiated by the recipient. Studies have confirmed that CAR T cells can relieve patients with post-transplant relapse without a flare of aGVHD. For recipient-derived CAR-T cell therapy, Park et al. (18) and Lee et al. (9) reported a total of 25 patients with no GVHD observed. For donor-derived CAR T cell therapy, Cruz et al. (22) and Kochenderfer et al. (23) reported a total of 38 patients without aGVHD flare.

From the above data, either recipient-derived or donor-derived CAR T cell therapy has good efficacy in the treatment of post-transplant relapse, with low risk of aGVHD. However, the reported data are focused on relapse after HLA-matched transplantation. Limited data of CAR T cells in relapse after haploidentical transplantation from China showed different results. Huang XJ et al. reported that out of 34 evaluable patients, 7 patients developed GVHD, with mild chronic GVHD in 1 case and aGVHD in 6 cases (2 grade II aGVHD, 3 grade III aGVHD and 1 grade IV aGVHD). The occurrence of GVHD had a poor effect on survival (24). A study by Zhang X et al. showed that 2 out of 43 patients with post-transplant relapse developed ≤ grade II aGVHD when receiving allogeneic CD19 CAR T cells (25). In another study focusing on CAR T cell therapy in post-transplant patients, the development of aGVHD was observed in 10 patients (66.67%), with 6 patients developed grade I-II of aGVHD, while 4 patients developed grade III-IV of aGVHD (26). In the authors’ report, out of 20 patients, 10 (50%) developed new-onset aGVHD after CAR T cell infusion. The log rank test showed that high aGVHD occurrence was related to the source of CAR T cells in the haploidentical transplantation group (*P* = 0.005).

According to the above studies, patients were likely to develop aGVHD 3 to 4 weeks after CAR T cell treatment. This may be related to the survival time of CAR T cells. aGVHD usually occurs in 1 month after lymphocyte infusion, equal to the survival time of CAR T cells *in vivo* (27). Therefore, the possibility of aGVHD is low. The occurrence of aGVHD is related to the dose of infused lymphocytes. The number of CAR T cells infused was between 10e^6^ and 10e^7^, which was less than the threshold of 10e^7^ for aGVHD (28). On the other hand, fludarabine and cyclosporine as GVHD prophylaxis in bone marrow transplants, may reduce the risk of CAR T-related aGVHD (29). Different CAR structures and different stimulation intensities also cause differences in aGVHD occurrence. Kawalekar et al. believed that CAR T cells with 4-1BB as a costimulatory molecule were less activated and did not often cause aGVHD (30). Tao Wang et al. used CAR T cells as DLI treatment, which significantly prolonged the survival period. They found a lower incidence of aGVHD than that of traditional DLI (31).

In essence, both aGVHD and CRS are inflammatory cascades. Nevertheless, aGVHD and CRS share similar symptoms in mild cases. Therefore, sometimes it’s difficult to distinguish between the two common complications. Clinically, CRS usually occurs within 1-14 days after CAR T cell infusion (32), while aGVHD can happen in 100 days after transplantation (13). CAR T-related CRS is believed to be caused by the activation of myeloid cells by highly activated T cells, and IL-6 released from myeloid cells has been shown to be an important contributor (33). Pre-use of IL-6 and IL-1 receptor antagonists can prevent CRS without compromising tumor remission, but only IL-1 receptor blockade can prevent neurotoxicity. IL-2 secreted by Th1 cells is the most important cytokine that initiates aGVHD (34). Animal experiments have found that in the first two days of aGVHD, the IL-2 produced by donor CD4+ T cells reaches the highest level. Mice injected with IL-2 can aggravate the degree of aGVHD (35). Another major cytokine is IFN-γ, with the peak level appears on the 7th day after transplantation (36). Therefore, cytokine detection can also distinguish aGVHD and CRS.

## Conclusion

In summary, the initial efficacy and safety of CAR T cell therapy for patients with relapsed B-ALL after HSCT were satisfactory. Relapse after CAR T cell therapy remains a big obstacle that influents patients’ long-term survival. The incidence of aGVHD was higher in patients with a previous history of haploidentical transplantation, making it a concern for CAR T cell therapy, especially for patients receiving allogeneic CAR T cells. The treatment of aGVHD after CAR T cell therapy is the same as the treatment of aGVHD after HSCT. We are conducting further research with a larger sample size so that more credible conclusions can be drawn.

## Data Availability Statement

The original contributions presented in the study are included in the article/supplementary material. Further inquiries can be directed to the corresponding author.

## Ethics Statement

The studies involving human participants were reviewed and approved by the Ethics Committee of the First Affiliated Hospital of Zhejiang University School of Medicine. Written informed consent to participate in this study was provided by the participants’ legal guardian/next of kin.

## Author Contributions

LD and YW contributed equally to the work. They collected patients’ data and wrote the manuscript together. RH, HZ, and LZ participated in the data collection and analysis. GW, WW, YL, and JS, as clinicians, participated in the patient’s diagnosis and treatment process, and provided valuable clinical data. HX, YZ, and AC were responsible for the preparation and quality inspection of CAR T cells. HH and YH as corresponding authors, guided the entire research and manuscript writing. All authors contributed to the article and approved the submitted version.

## Funding

This work was supported by grants from the National Natural Science Foundation of China (81730008, 81970137), the Key Project of Science and Technology Department of Zhejiang Province (2018C03016-2, 2020C03G2013586), and the Key Research and Development Program of Zhejiang Province (2019C03016).

## Conflict of Interest

Authors YZ and AC were employed by Shanghai YaKe Biotechnology Ltd..

The remaining authors declare that the research was conducted in the absence of any commercial or financial relationships that could be construed as a potential conflict of interest.

## Publisher’s Note

All claims expressed in this article are solely those of the authors and do not necessarily represent those of their affiliated organizations, or those of the publisher, the editors and the reviewers. Any product that may be evaluated in this article, or claim that may be made by its manufacturer, is not guaranteed or endorsed by the publisher.
